# Chloroplast Genome Engineering: A Plausible Approach to Combat Chili Thrips and Other Agronomic Insect Pests of Crops

**DOI:** 10.3390/plants12193448

**Published:** 2023-09-30

**Authors:** Mallesham Bulle, Vijay Sheri, Mahender Aileni, Baohong Zhang

**Affiliations:** 1 Agri Biotech Foundation, Agricultural University Campus, Rajendranagar, Hyderabad 500030, India; 2 Department of Biology, East Carolina University, Greenville, NC 27858, USA; sheriv22@ecu.edu; 3 Department of Biotechnology, Telangana University, Dichpally, Nizamabad 503322, India; ailenidrmahender@gmail.com

**Keywords:** chloroplast engineering, CRISPR, double-stranded RNA, mini chromosomes, RNA-binding proteins, thrips

## Abstract

The world population’s growing demand for food is expected to increase dramatically by 2050. The agronomic productivity for food is severely affected due to biotic and abiotic constraints. At a global level, insect pests alone account for ~20% loss in crop yield every year. Deployment of noxious chemical pesticides to control insect pests always has a threatening effect on human health and environmental sustainability. Consequently, this necessitates for the establishment of innovative, environmentally friendly, cost-effective, and alternative means to mitigate insect pest management strategies. According to a recent study, using chloroplasts engineered with double-strand RNA (dsRNA) is novel successful combinatorial strategy deployed to effectively control the most vexing pest, the western flower thrips (WFT: *Frankliniella occidentalis*). Such biotechnological avenues allowed us to recapitulate the recent progress of research methods, such as RNAi, CRISPR/Cas, mini chromosomes, and RNA-binding proteins with plastid engineering for a plausible approach to effectively mitigate agronomic insect pests. We further discussed the significance of the maternal inheritance of the chloroplast, which is the major advantage of chloroplast genome engineering.

## 1. Introduction

The global demand for food is projected to rise from 35% in 2010 to 56% by the year 2050 [1]. However, agronomic productivity is severely affected by environmental factors, as well as infestations of insect pests and diseases. Thus, insect pests alone contribute to an approximately 20% reduction in crop productivity every year at a global scale [2]. Thrips are among the most significant members of a broad group of agronomic invasive pests. Over 150 crops, including peppers, cotton, strawberries, citrus, tomato, and many other crops, are known to have invasive pests’ threat [3]. Because of their broad range of hosts, tiny size, and rapid reproduction and growth, these pests show devastating effect in their productivity not only in fields, but also on crop production in other conditions, such as in greenhouses [4]. Chili pepper (*Capsicum* spp.) is widely cultivated and is among the most popular horticultural crops globally. The fruits of different Capsicum species are largely consumed due to their nutritional and pharmaceutical significance. Among different chili growing countries, India holds the top position as the world’s leading producer, accounting for 36% (0.45 million tons annually), followed by China, Thailand, Ethiopia, and Indonesia [3]. Chili pepper plants are most vulnerable to native and invasive pests during their growth and development. The genetic mechanisms that govern thrips’ control, feeding behaviors, and pesticide resistance remain largely unknown. Obviously, the first line of protection against insect pests is the usage of chemical pesticides due to their effective means of insect pest control. However, deployment of such noxious chemical pesticides always has threatening effect on human wellbeing and the sustainability of the environment [5].

The chili infesting pests exhibit lot of diversity, and over 293 species of insects and pests were found to be severely damaging the chili crops in both field and storage conditions [6]. Around 16 thrips species were recognized for causing damage to *capsicum* plants worldwide [7]. Among them, *Frankliniella occidentalis* is the prevalent thrip of *capsicum* in Europe [8], whereas *T. parvispinus* is the most common insect pest of the same crop in Indonesia, Malaysia, the Philippines, Thailand, and Taiwan or China [3]. *T. parvispinus* is a polyphagous pest reported for the first time in the Telangana State chili growing areas of India and has been recorded as a serious devastating insect pest of *capsicum* worldwide [9]. *T. parvispinus* is a notorious invasive pest originating from Thailand [10] and is known to act as a viral vector to spread diseases on several horticultural crops, including chili peppers [9]. Moreover, there are over 20 insect species infesting Indian chili peppers; of these, thrips (*Scirtothrips dorsalis*), mites (*Polyphagotarsonemus latus*), and aphids (*Aphisgosypii* and *A. craccivora*) are the most harmful pests [3]. The Asian Vegetable Research and Development Committee considered that *S. Dorsalis* is among the most significant thrips in terms of affecting the annual chili yield [3]. The range of yield loss attributable to *S. dorsalis* alone is between 61 and 74%. Within sucking pests, the chili thrips *S. dorsalis* is regarded as the most devastating pest [11].

In chili pepper, estimated losses caused by thrips were between 50 and 90% globally [12]. Additionally, climate change and the rapid adaptability of thrips are wreaking havoc on horticultural crops worldwide but are particularly prevalent in developing nations, such as in India. During seasons of dry weather, thrips reproduce at an accelerated rate, causing yield losses of 30 to 50% in India [4,13]. Hence, an increase of one degree Celsius in the mean temperature is attributed to increased yield losses from 10% to 25% [14]. In this paper, the devastating effect of an insect pest (thrips) on the annual production of the chili pepper sps. is an example of how insect pests are responsible for a devastating impact on agronomic food productivity. The yield and economic losses due to the agronomic pests highlight the need to develop cutting-edge, eco-friendly, and cost-effective mitigation approaches against insect pests, including thrips. The opinions concluded here in this paper on various biotechnological research methods may facilitate the researchers’ search for a plausible solution to effectively control different agronomic pests.

### 1.1. Feeding Mechanisms and Response of Plants to Thrips Invasion

In recent decades, significant attempts have been dedicated to the advancement of several crops, which shows enhanced resistance to insect pests and viruses. However, resistance to thrips feeding has recently garnered increased attention [15,16]. Understanding the nutritional requirements and feeding mechanism of thrips is important for developing effective control strategies to manage different agronomic pests, including invasive thrips and the tospoviruses that it vectors [16]. Thrips generally harm crops by scraping the outer layer (epidermis) and sucking the cell sap from young leaves, making them curl up and become smaller, thus, affecting growing shoots [17]. Depending on the chemical constituents and appropriate food source, the host plant accepts thrips for feeding [18]. When thrips are allowed to infest, adult thrips extend their antennae and labial sensilla over a putative host plant surface to check for the right chemical and physical stimuli for feeding [16]. 

Apart from causing crop loss, thrips indirectly harm crops by transferring plant viruses because they tend to act as viral vectors [19]. For example, western flower thrips (WFT) acts as vector for seven species, including Orthotospovirus [20]. Furthermore, studies have shown that when plants are infested with thrips species, it triggers the production of jasmonic acid (JA), which is essential for initiating plant defense mechanisms. For example, most genes that are activated in *Arabidopsis* when WFT feeds on it are responsive to JA, resulting in elevated JA levels [21]. These defense responses subsequently influence how WFT interact with these potential host plants [22]. 

The approach typically employed in the investigation of host plant resistance (HPR) involves phenotypic screening of the germplasm for resistant genotypes [23,24,25]. Advances in genome editing tools, such as CRISPR/Cas, have provided a significant boost in the breeding strategies and the advancement of our understanding of plant–pest defense mechanisms by connecting genotype–phenotype relationships [26,27,28].

### 1.2. Plastid Inheritance and Advantages of Chloroplast Engineering

The chloroplast is a pigmented semi-autonomous organelle in plants, having a very large copy number of chloroplast DNA (cpDNA) per leaf cell. Thus, among plastids, the chloroplast has a high potential for transgene expression, and as it contains multiple circular chloroplast genomes, it has the tendency to express the genes at the same time. Furthermore, the chloroplast genomes are inherited maternally and are, thus, inaccessible to sexual recombination [29]. The commonly stated reason for maternal inheritance of plastid-associated traits in plants is due to the lack of plastids in the generative cells of pollen [30]. Recent investigations have shown that plastid inheritance is controlled by very intricate gene–environment interactions, despite the fact that the basic mechanisms of maternal inheritance are still unclear [31]. The chloroplast is a plastid, whose genetic engineering assures gene containment, which further alleviates concerns about low levels of transgene expression, gene silence, positional and pleiotropic effects, and the inclusion of vector sequences in transformed plant genome [32,33]. Thus, plastid transformation is more advantageous than nuclear transformation in many ways. The most important one, maternal inheritance of the plastid genome, which effectively prevents genes from being released via pollen in most crops. This mechanism helps minimize the dissemination of transgenes in the environment, mitigating concerns related to genetically modified organisms (GMOs) [34]. Second, the abundance of cpDNA copies leads to elevated expression of exogenous genes and the subsequent accumulation of proteins they encode [35]. Thirdly, due to the utilization of homologous recombination (HR) for transgene integration into a designated target site during plastid transformation, this approach effectively mitigates the gene silencing that can result from position effects [36]. These critical hallmarks represent that chloroplast engineering is a feasible alternative to conventional nuclear transgenic methods, primarily because of the improved transgene containment of transplastomic plants [36]. Nevertheless, the transplastomic technology, a way towards eco-friendly agriculture, remains largely unavailable for the majority of staple crops, including cereals. However, plastid transformation is always the favorite means of producing designer crops for improved crop yield and/or resistance, and hopes to enable sustainable crop production with minimal risks and societal concerns [37].

## 2. Biotechnological Approaches to Combat Invasive Pests

Genome editing, plastid-mediated gene silencing, and gene transfer are the several biotechnological methods used to manipulate the genetic elements of plant systems to control and possibly eliminate insect pests, including invasive thrips. Host plant resistance is one of the crucial approaches to control invasive pests in modern agriculture [38,39]. Manipulating the expression of genes, CRISPR reagents, and RNPs in plants, are widely used to improve their endurance for insect damage [40]. During the last 30 years, numerous insect-resistant cultivars have been developed by various research institutions deploying either traditional or advanced biotechnological approaches [41].

### 2.1. Deployment of Homology-Based Recombination in Chloroplasts

In plastid engineering via homologous recombination (HR) between the transformed vector’s targeting regions and the wild-type plastid DNA, foreign DNA is integrated into the plastid genome at a predefined and desired position [42]. Thus, HR enables the insertion of transgene sequences responsible for position effects in nuclear transformation studies [36]. In recent years, scientists have been attempting to engineer chloroplast genomes with desirable economic and agronomic characteristics. Developing insect resistance in crop plants via plastid transformation is one of the most remarkable achievements for crop improvement. For instance, expression of the *beta-glucosidase* (*Bgl-1*) gene in tobacco chloroplasts resulted in 160-fold higher phytohormone levels and resistance to whiteflies and aphids [43], whereas expressing the *Btcry1Ab* gene in soybean chloroplasts resulted in resistance to caterpillar (*Anticarsiagemmatalis*) [44]. Likewise, engineering tobacco plastids with *Bt-Cry9Aa2* and *cry2Aa2* genes resulted in enhanced resistance to potato tuber moth (*Phthorimaeaoperculella*) and moths (*Heliothisvirescens, Helicoverpazea, Spodoptera exigua*) [45,46]. However, the frequency of HR events greatly relies on the vector sequences being similar to the target integration site by at least 121 base pairs [47]. To improve transformation efficiency, the promoter, regulatory elements (5′ and 3′ UTRs), and the location of insertion in the plastid genome should be designed carefully. Moreover, the excessive production of foreign proteins in chloroplasts might impose a metabolic drain on transplastomic plants, resulting in the development of severe mutant phenotypes [48,49]. Consequently, various inducible expression systems in plastids to counter adverse consequences have been developed. These include utilizing the bacterial *lac* repressor and *lac* operator [50], ethanol-inducible T7 RNA polymerase [51], and the theophylline-inducible riboswitch [50,52].

### 2.2. Plastid-Mediated RNAi Interference (PM-RNAi)

There are different biotechnological crop protection strategies available to limit insect infestations. For over a decade, RNA interference (RNAi)-based tactics have been demonstrated to be a more efficient strategy than approaches based on the expression of long double-stranded RNA (dsRNAs) or hairpin RNAs (hpRNAs), encoded by transgenes of the host (plant) nuclear genome [53,54]. dsRNAs are double-stranded RNA molecules, whereas hpRNAs are self-complementary RNAs. In RNAi, the ribonuclease DICER identify and converts double-stranded viral RNAs into shorter 20–24-nucleotide (nt)-length RNA duplexes known as siRNAs or miRNAs. Subsequently, these siRNAs are incorporated into the RNA-induced silencing complex (RISC) to cut, destabilize, or hinder the translation of homologous mRNAs [55,56]. In a nutshell, plants express dsRNAs to target pest genes; the process begins upon their consumption via the gut cells of pests; later, dsRNA is processed to produce small interfering RNAs (siRNAs). This mediates the degradation of messenger RNA (mRNA), resulting in the knockdown of the target gene in the insect pest, the silencing of which is detrimental to the development and survival of that insect pest [57]. Unfortunately, in transgenic plants, endogenous RNAi machinery converts dsRNA expression into siRNA, which reduces the dsRNA interference effect on the insect’s targeted mRNA [57,58]. In contrast, plastid-mediated RNAi interference (PM-RNAi)-based approaches could be an alternative strategy to effectively mitigate insect infestation in crops.

Various researchers have attempted to create transplastomic plants resistant against insect pests, wherein the tendency of high protein expression levels of genes by chloroplast genomes are exploited (Table 1). For instance, transplastomiccry1A(c) plants accumulated significant amounts of total soluble cry1A(c) protein, which demonstrated strong resistance to diverse pests for tobacco (*Nicotiana tabacum*) [59]. Transplastomic plants of soybean and poplar also showed high resistance to pests [44,60]. Zhang and colleagues [61] developed effective pest control strategy that include the expression of long dsRNA in potato (*Solanum tuberosum*) plastids to silence the essential gene *β-Actin* of the insect pest *Leptinotarsa decemlineata*.

**Figure 1:**
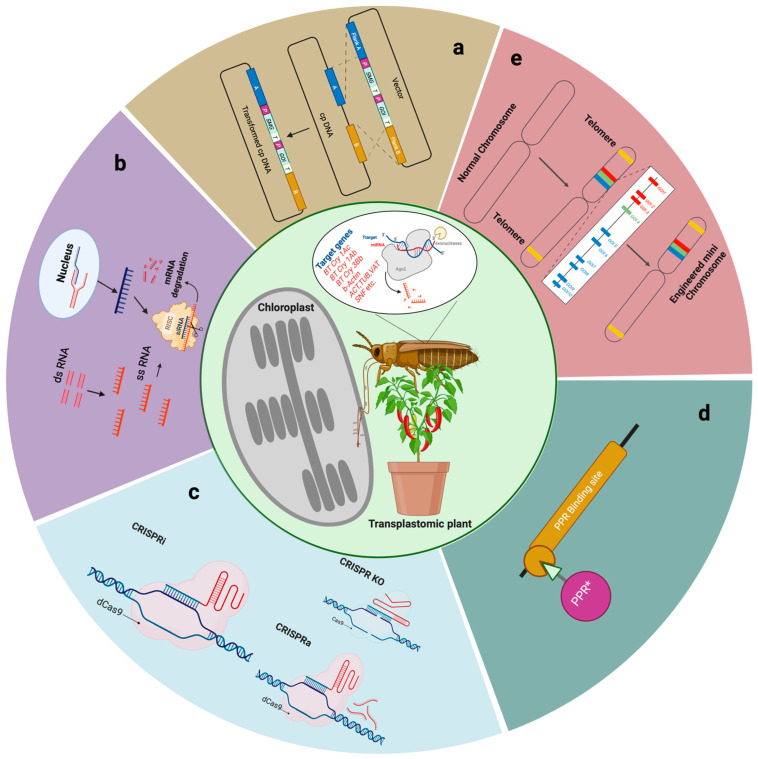
Various biotechnological approaches to combat agronomic insect pests. Chloroplast engineering strategies to develop insect pest-resistant crops using various molecular techniques, such as (**a**) homology-based recombination in chloroplasts, (**b**) chloroplast engineering mediated by RNA interference, (**c**) engineering chloroplasts by a CRISPR/Cas editing system, (**d**) RNA binding proteins (* designed pentatricopeptide repeat, PPR) to regulate gene expression in chloroplasts; (**e**) replicating plant mini chromosomes for chloroplast genome editing. When thrips feed on transplastomic plant mesophyll cells, they devour cellular content (sap) including chloroplasts, leading to their high mortality. This is due to designed molecules that inactivate/interfere with the target mRNAs necessary for their survival and development processes. CRISPR/Cas, clustered regularly interspaced short palindromic repeats/CRISPR-associated protein; CRISPRko, (knock out); CRISPRi, (interference); CRISRPa, (activation); ssRNA, single-strand RNA. Figure created with BioRender.com. https://www.biorender.com (accessed on 21 September 2023).

When feeding on a host plant, thrips typically consume cellular contents (sap), including chloroplasts. It is shown that engineering chloroplast genomes with long dsRNAs targeting insect housekeeping genes is an effective management strategy (Figure 1). Recently, the same tactic was employed to control the WFT [57], wherein the expressed dsRNAs or hairpin RNAs (hpRNAs) were targeted using the genes of actin (*ACT*), tubulin (*TUB*), vacuolar ATPase catalytic subunit B (*VAT*), and the endosomal sorting complex of transport III subunit SNF7 (*SNF*). These four targeted genes have critical roles in the growth and survivability of pests [61,69,70,71]. Another study also demonstrated that the plastid-mediated expression of dsRNAs has immense potential to control both chewing and non-chewing pests [58].

RNAi generally targets a specific pest, but the inserted dsRNA may also function on other non-targeted insects. Thus, RNAi technology raises such severe concerns due to its off-target and non-target effects. Sequence homology between siRNAs and non-target genes, particularly in the 3′ untranslated regions (UTR), is found to be crucial for off-target effects [72]. However, this limitation can be circumvented by explicitly selecting the target region.

### 2.3. Engineering Chloroplasts Using CRISPR/Cas9 and Integrated Genome Editing Systems

CRISPR/Cas9 is a swiftly advancing RNA-guided genome editing technology that has been harnessed for investigating gene functions in a variety of cells and organisms, including plants, wherein it is restricted to editing and studying the role of genes, particularly nuclear genes [73]. This might be due to the difficulty in transportation of the Cas9 protein and gRNA into the chloroplast genome and their simultaneous expression; thus, its applicability to examine chloroplast gene editing is low [74]. Plants’ chloroplast genome sequences were thought to resemble those of *cyanobacterial* progenitors in certain aspects [75]. Thus, due to the similarity between the genomes of *cyanobacteria* (green microalgae) and chloroplasts, researchers tried to alter the microalgae’s chloroplast genes. However, the production of transformants has remained difficult due to the toxicity linked to continuous expression of Cas9 in the green microalga *Chlamydomonas reinhardtii* [76]. Yoo et al. [77] have demonstrated “Edit Plasmids”, a novel CRISPR-based chloroplast genome editing method in *C. reinhardtii*. Without deploying a CRISPR/Cas9 system, Kang et al. [74] demonstrated a chloroplast editing technique for the generation of transplastomics. They created a high-fidelity DddA-based cytosine base editor (DdCBE), a precision mitochondrial DNA editing plasmid. This plasmid, along with the 424 transcription activator-like effector and 16 expression plasmids, was designed to improve the efficiency of point mutations in lettuce and rapeseed calli chloroplast genomes. However, such studies failed to determine the effectiveness of the DdCBE system in chloroplast editing.

The efficiency of CRISPR/Cas9 has been reported to excel in editing the nuclear genome of plant species [78]. However, it seems that it is difficult to edit plastid gene because it requires researchers to transfer the CRISPR/Cas9 reagents into the plastid first. The essential step for HR in chloroplast transformation is the induction of double-stranded DNA breaks (DSBs) in the plastid genome [79], which assists the desired editing location by the CRISPR nuclease Cas9. In a recent study, Yoo et al. [77] performed genome editing in the plastids of *Chlamydomonas* through the introduction of two plasmids. One plasmid encompassed a combination of a guide RNA (gRNA) and Cas9 protein, while the other plasmid carried the donor DNA fragment intended for integration at the DSB site generated by the first plasmid. In this approach, the Cas9/gRNA cassette was inserted under the regulation of the potent chloroplast *psbA* promoter, leading to the transformants with the integration of the donor plasmid at the desired integration site. These findings suggest that DNA DSB breaks do facilitate donor DNA integration in the chloroplast genome.

Although CRISPR/Cas9 technology is promising, various challenges, such as delivering guide RNA and Cas proteins into organelles, etc., still need to be overcome [80]. Furthermore, CRISPR-Cas9 and related base editors, renowned for their precision in modifying nuclear DNA, fall short when it comes to editing organellar DNA [80]. In order to solve the challenges associated with organellar DNA editing, a recent work conducted by Nakazato et al. [81] demonstrated the effective application of a method developed for precise base editing of the *Arabidopsis* chloroplast genome. This was accomplished by employing bacterial cytidine deaminase coupled with transcription activator-like effector nucleases (TALEN). This study has revealed the occurrence of homoplasmic substitutions, specifically changing Cs to Ts at targeted locations, in T1 generation plants. 

Furthermore, Mok et al. [82] recently reported heritable A-to-G edits in chloroplast DNA through TALE-linked deaminases (TALEDs). Using this strategy, they targeted the three (*psbA, psaA*, and *rrn16*) chloroplast genes in lettuce protoplasts, resulting in 3.5% C-to-T conversions and an impressive 25–51% A-to-G conversion efficiency. Stable *Arabidopsis* lines were also established, targeting chloroplast *psaA*, *rbcL*, and *rrn16S* genes, revealing that these TALED-mediated edits in chloroplast DNA remained stable and were heritable in subsequent generations. These investigations presents compelling evidence supporting the feasibility of targeted base editing within the plastid genome. 

### 2.4. RNA-Binding Proteins Regulate Gene Expression in Chloroplasts

RNA-binding proteins are pivotal in performing critical biological processes, including the regulation of gene expression and the creation of scaffolds for structural changes at the macromolecular level [83]. The best-characterized RNA-binding proteins are pentatricopeptide repeat proteins (PPR), and they are prevalent in terrestrial plants. Interestingly, the majority of PPR proteins act as RNA mediators, which influence a variety of organellar metabolic processes [84]. Although encoded by nuclear genes, PPR proteins function in a sequence-specific manner within chloroplasts and mitochondria [85]. Numerous scientists have engineered pentatricopeptide repeat proteins (dPPRs) to modulate RNA levels. Coquille and team [86] effectively created artificial PPR domains by leveraging conserved residues within PPRs. The dPPR domains were found to be extremely soluble and showed sequence-specific binding to target RNAs. A few years later, Yin’s group [87] developed several synthetic PPR proteins that have the tendency to bind specific regions of targeted single-stranded RNAs (ssRNAs). Their study provided detailed and specific binding models for dPPR repeats, and further established the groundwork for the advancement of RNA manipulation technologies [87]. So far, limited in vivo dPPR experiments have been carried out. In a study, dPPR protein expression via nuclear transgenesis was carried out, wherein around a 40-fold enhancement in the expression of foreign plastid genes was reported [88]. In another study, Barkan’s lab effectively manipulated a dPPR protein in *Arabidopsis*, demonstrating its capability to bind particular mRNA sequences found in chloroplasts. This study was pioneered such that the synthetic dPPR protein functions in vivo to locate targeted RNA [88]. Meanwhile, a functional complementation study performed by [89] demonstrated that the synthetic dPPR protein exhibits a high degree of specificity in vivo when binding to its anticipated target mRNA. Moreover, they successfully substituted a natural PPR protein with the synthetic variant without affecting the processing of *rbcL* mRNA. These findings substantiate that dPPR proteins are the right candidates (as they are artificially generated and manipulated) to mimic the functions of native PPR proteins, emphasizing the different ways of regulating when, where, and the expression level of chloroplast genes. The diverse array of dPPR proteins, each with distinct RNA binding sites, serve as valuable tools for harnessing the distinct characteristics of chloroplast gene expression.

### 2.5. Replicating Plant Mini Chromosomes for Chloroplast Genome Engineering

Mini chromosome technology favors the stacking of genes on the same chromosome, which reduces the possibility of new traits segregation. These are also called “artificial chromosomes”. In plants, artificial chromosomes were initially studied in 2006, and a subsequent study by Yu and colleagues [90] successfully introduced a 2.6 kb segment of *Arabidopsis*-like telomere repeats into maize (*Zea mays*) using the *Agrobacterium* transformation method. Telomere-based chromosomal truncation (TMCT) is used in the formation of artificial mini chromosomes. Mini chromosomes are found to segregate independently of host chromosomes, which has opened the way for quicker plant breeding [91,92]. Moreover, artificial chromosomes operate similarly to natural chromosomes, exhibiting typical functionality without pairing with existing chromosomes or adversely impacting plant development [90]. 

In addition, replicating mini chromosomes offers an attractive research tool for transgene insertion and expression in subcellular compartments of plant cells, especially chloroplasts [93]. The transgene is amplified with the aid of a helper protein in this method of plastid engineering, wherein significant amounts of protein accumulation and expression of foreign genes were achieved. Such methodologies ensure the stability of transgene amplification and transmission of genetic material to progeny through maternal inheritance, avoiding the integration of foreign DNA into the plastome. However, the implications of plant mini chromosomes (PMCs) in plants are unknown due to several potential problems; the bottom-up approach with epigenetic influence generally fails to form an active centromere, which is necessary for sister chromatid segregation during anaphase [92]. While the top-down strategy is based on TMCT, it also confronts many problems, notably the truncation of normal chromosomes, which leads to the loss of some chromosome regions; this has been found to be detrimental to plant growth and viability [94]. Thus, the technological limitations of PMCs restrict their future development and applications [95].

## 3. Conclusions and Perspectives

Insect pests not only directly cause significant damage to agriculture crops but also indirectly act as carriers of deadly viruses. Such a biological invasion has a negative impact annually on the economics of crop productivity and, thus, affects the native ecosystem and promotes biodiversity loss. The advent of transgenic technology has increased the possibilities of transferring insect-resistant genes from a non-plant origin and heterologous species to plants for crop improvement. For instance, insect-resistant plants for commercial use have been engineered by introducing insecticidal genes sourced from bacterial organisms [66,96,97,98]. Nonetheless, in numerous countries, the full potential advantages of this technology have not been realized due to overly restrictive regulations [99]. While chloroplast engineering is an environmentally beneficial method, it does not lead to gene pollution, as nuclear plant transformation research has been stated to do. This is because when the desired gene is incorporated into chloroplast and inherited maternally (confined to the gynoecium of the flower/gene containment), pollen cannot escape into the environment.

The homologous recombination method encompasses the possibility of foreign DNA integration at precise locations of plastid DNA. For the advantage of chloroplast engineering, such a method needs to be further investigated. The CRISPR-associated component Cas9 protein is widely deployed in the process of precise genome editing studies [100]. A newly identified or modified endonuclease variants, such as Cas12, refs. [100,101,102] was used to overcome the problems encountered due to their size and to facilitate enhanced stability and targeted mutagenesis. These avenues are tempting to deploy such identified small and stable endonucleases for extending gene editing in sub-cellular locations, especially in chloroplast genome editing. Moreover, recent advancements have been made in base editing in plant organelles [74,103], demonstrating the possibility of precise DNA-free base editing in organelles.

Expression of metabolic pathway genes in chloroplasts with modified volatile plant chemicals can be alternative applications for controlling invasive pests, like *Thrips parvispinus* (Karny). Volatile plant chemicals are produced by the conversion of tryptamine to serotonin catalyzed by the enzyme encoded by the *CYP71A1* gene. In rice plants, the production of serotonin has been triggered by insect infestation. Suppressing serotonin biosynthesis by knockdown *CYP71A1* gene in rice plants conferred resistance to major insect pests [49].

Combining RNAi with plastid engineering could be the most probable strategy for engineering resistance against agronomic pests. A successful combinatorial study demonstrated control of the most vexing pest, *Frankliniella occidentalis* [57].

Chloroplast engineering has hallmarks, such as molecular farming, but it is still in its nascent stage for crop plants, like chili pepper, cereals, and pulses. In several crop plants, such as chili peppers, the advancement of insect-resistant crops is in nascent stage. Poor in vitro regeneration efficiency during plant tissue culture is another major constraint for any plastid-mediated transformation protocol. Overexpression of developmental regulatory genes, including growth-regulating factors (GRFs) and GRF-interacting factors (GIFs), Baby Boom (*BBM*), and Wuschel2 (*WUS2*), has been shown to improve plant regeneration, thus, resulting in an increase in nuclear transformation efficiency [104,105,106], but their effect on plastid in vitro regeneration efficiency remains to be demonstrated in recalcitrant species, such as chili pepper.

In this short review, we proposed that combining a RNAi approach with plastid engineering would be an effective combinatorial strategy for engineering resistance against agronomic pests, annually and at a global scale. These pests are of serious concern as they cause major ecological and economic damage to crop plant productivity. At the same time, as the quick deep sequencing technology develops [107], more genes will be found to use in plastid engineering against pests for long-term environmental sustainable development.

## Figures and Tables

**Table 1:** List of potential target genes engineered into crops by plastid transformation.

Gene Name	Function	Insect Species/Order Investigated	Molecular Approach	Host	References
*Thrips palmiUHRF1BP1*	Mortality and to impair virus transmission	Thrips palmi (*Thysanoptera: Thripidae*)	Silencing	*Thrips palmi*	[62]
*Thrips palmi PFAS*					
Western flower thrip Actin (*ACT*)	Mortality of *western flower thrip*	Western flower thrip (WFT, *Frankliniella occidentalis*)	Silencing	*Nicotiana tabacum*	[57]
Western flower thrip Tubulin (*TUB*)					
Western flower thrip Catalytic subunit B of vacuolar ATPase (*VAT*)					
Western flower thrip Endosomal sorting com-plex for transport III subunit *SNF7 (SNF)*					
*Phenacoccussolenopsis* *v-ATPaseA*	Efficient control of leaf-chewing and lacerate-and-flush feeding insects	Brown marmorated stink bug (BMSB) (*Halyomorphahalys*) Madeira mealybug (MMB) (*Phenacoccusmadeirensis*) Colorado potato beetle (CPB) (*Leptinotarsa decemlineata*)	Silencing	*Solanum lycopersicum*	[63]
*MyzuspersicaeMpDhc64C,* *Helicoverpaarmigera* *NDUFVII*	Efficient control of sap-sucking pests	*Myzuspersicae, Acyrthosiphonpisum,* *Adelphocorissuturalis,* *Rhopalosiphumpadi,* *Nilaparvatalugens*	Silencing	*Nicotiana tabacum*	[58]
*Spodoptera littoralis 102 (Sl102)*	1.Reduced food consumption of larvae 2. Impairment of hemocyte-mediated encapsulation response 3. Enhanced biopesticide activity	*Spodoptera littoralis*	Silencing	*Spodoptera littoralis*	[64]
Phenolic glucoside malonyltransferase gene *BtPMaT1*	Enhanced impeding the whiteflies’ capacity for detoxification	Whitefly (*Beniciatabaci)*	Silencing	*Solanum lycopersicum*	[65]
*Beta-glucosidase (Bgl-1)*	Elevated phytohormone levels and conferred resistance to whiteflies and aphids	Whitefly and aphids	Overexpression	*Nicotiana tabacum*	[43]
*Cry1Ab*	Resistance to *Caterpillar*	*Caterpillar (Anticarsiagemmatalis*	Overexpression	*Glycine max*	[66]
*Cry9Aa2*	Resistance to potato tuber moth	Potato tuber moth (*Phthorimaeaoperculella*)	Overexpression	*Nicotiana tabacum*	[46]
*cry2Aa2*	Resistance to moth	*Heliothisvirescens, Helicoverpazea, Spodoptera exigua*	Overexpression	*Nicotiana tabacum*	[45]
*β-actin encoding gene of whitefly ACTB*	Control of sap-sucking pest Whitefly	*Bemisiatabaci*	Silencing	*Nicotiana tabacum*	[58]
*cry1Ab*	Enhanced resistance to *Plutella xylostella*	*Plutella xylostella*	Overexpression	*Brassica oleracea*	[67]
*cry1C*	Improved toxicity to *Hyphantria cunea* and *Lymantria dispar*	*H. cunea and L. dispar*	Overexpression	*Hybrid Popular* *(P. davidiana × Populus bolleana)*	[68]

## Data Availability

The data presented in this study are available in this manuscript.
